# Supplementary material for: Intact-Cell MALDI-ToF Mass Spectrometry for the Authentication of Drug-Adapted Cancer Cell Lines
Source: Cells. 2019 Oct 2;8(10):1194. doi: 10.3390/cells8101194 (PMC6830094; doi:10.3390/cells8101194)
Supplement: Supplementary file 1 [file cells-08-01194-s001.pdf]

Figure S1

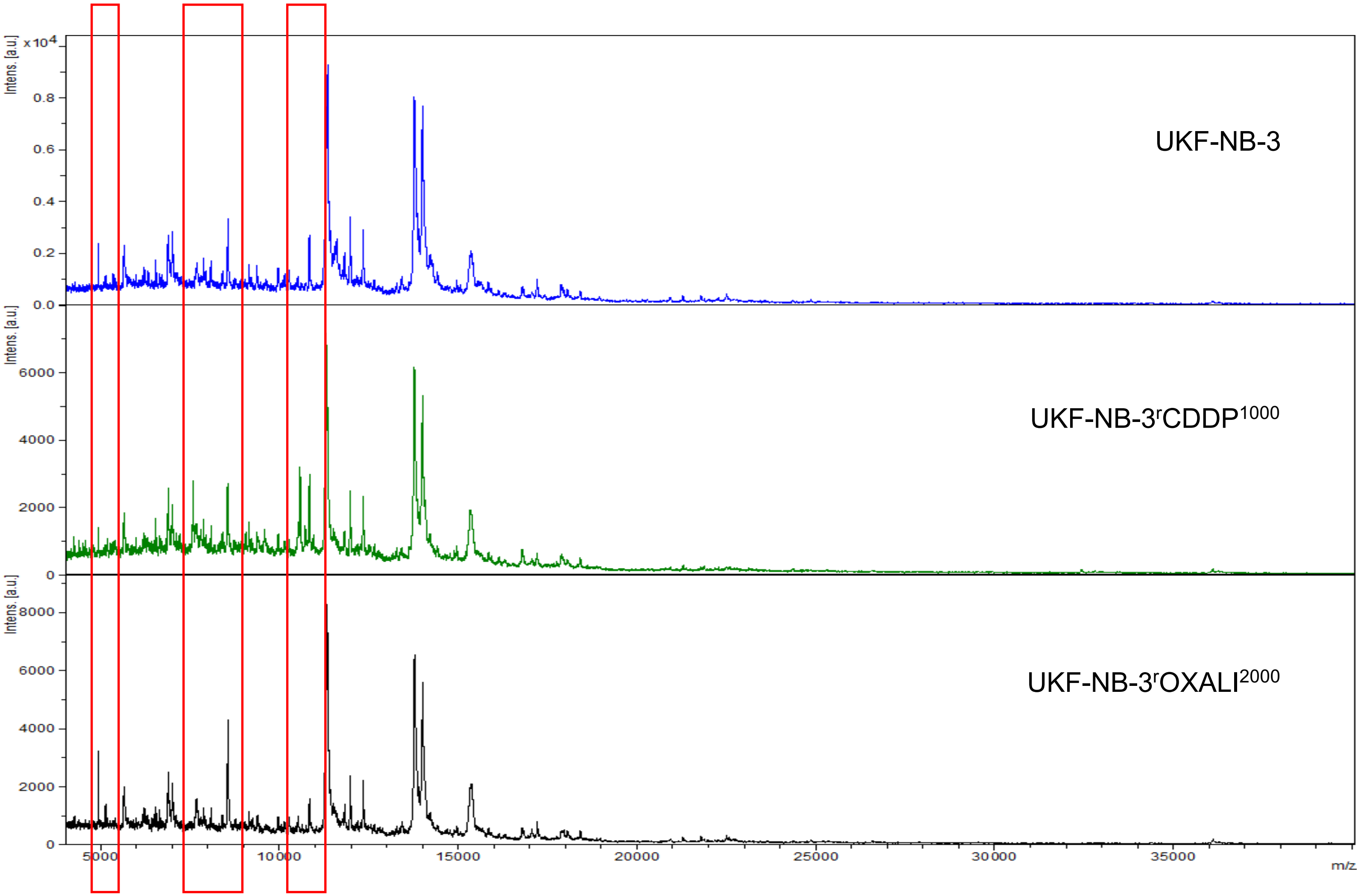

Figure S1. Representative intact cell MALDI-ToF mass spectrometry analysis spectra of the cell line UKF-NB-3 and its drug-adapted sublines. Some characteristic differences are highlighted in the red boxes.

Figure S2

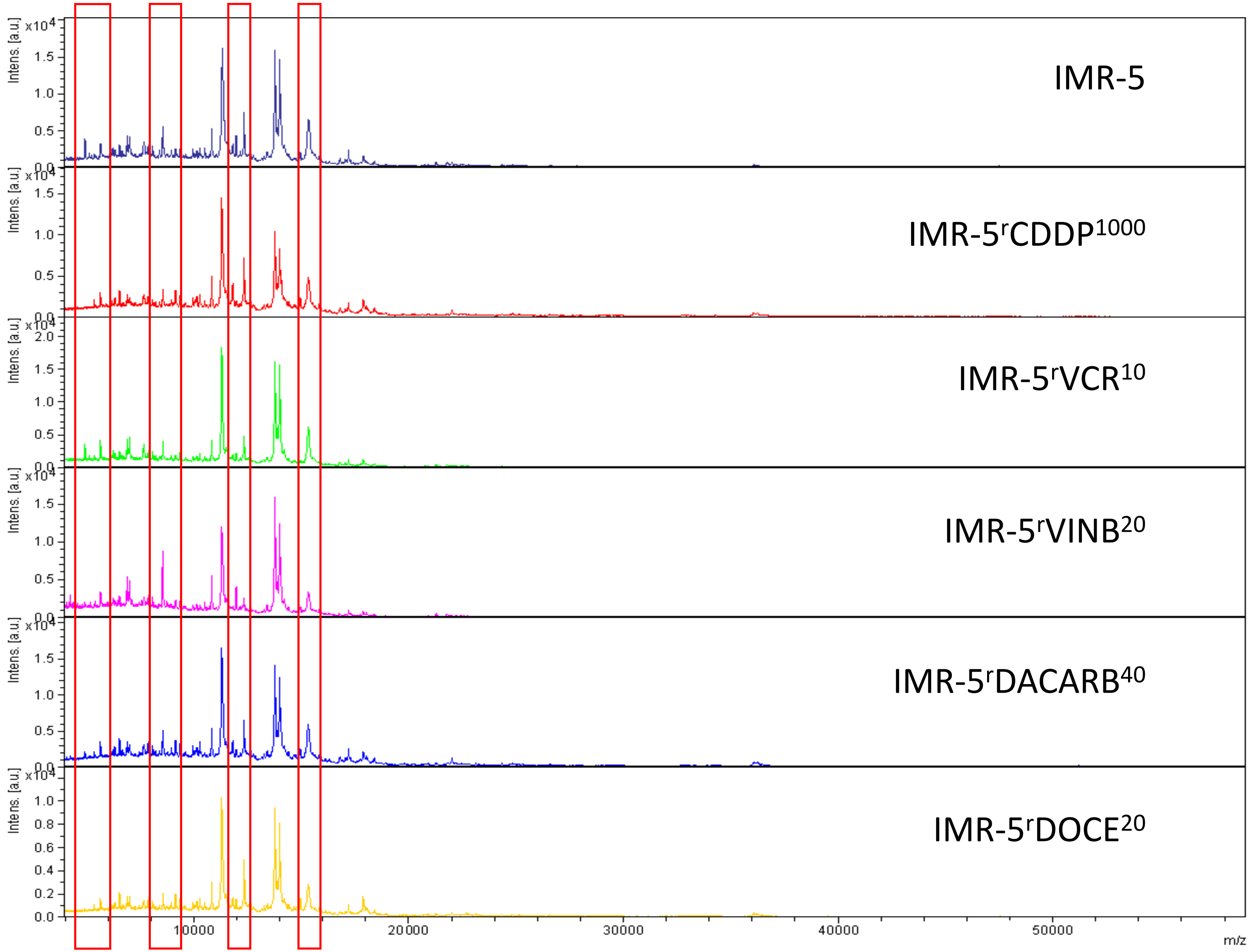

IMR-5
IMR-5rCDDP1000
IMR-5rVCR10
IMR-5rVINB20
IMR-5rDACARB40
IMR-5rDOCE20
Intens. [a.u.]
x10^4
m/z
10000
20000
30000
40000
50000

Figure S2. Representative intact cell MALDI-ToF mass spectrometry analysis spectra of the cell line IMR-5 and its drug-adapted sublines. Some characteristic differences are highlighted in the red boxes.

Figure S3

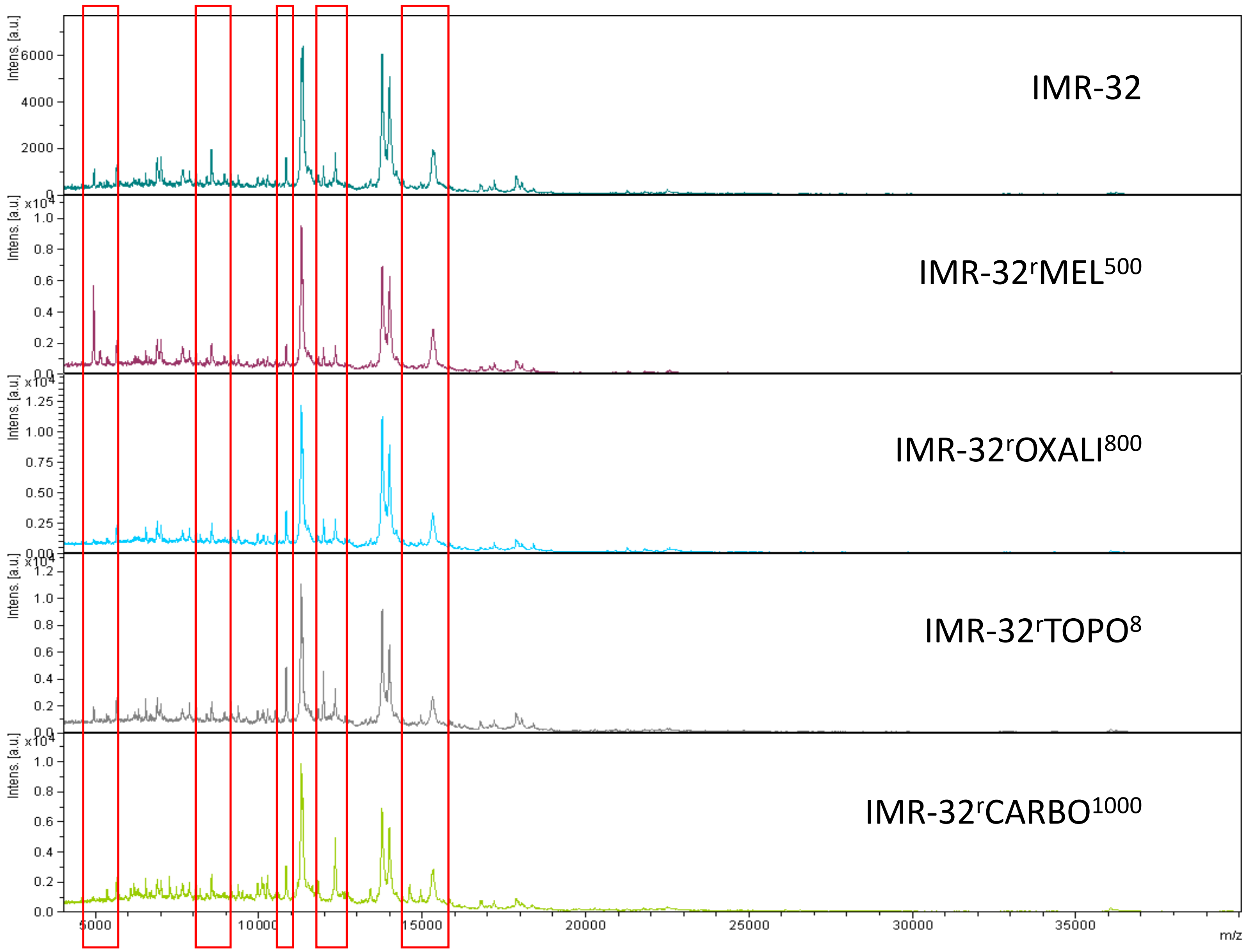

IMR-32
IMR-32rMEL500
IMR-32rOXALI800
IMR-32rTOPO8
IMR-32rCARBO1000
Intens. [a.u.]
x10^4
m/z
5000
10000
15000
20000
25000
30000
35000

Figure S3. Representative intact cell MALDI-ToF mass spectrometry analysis spectra of the cell line IMR-32 and its drug-adapted sublines. Some characteristic differences are highlighted in the red boxes.

Figure S4

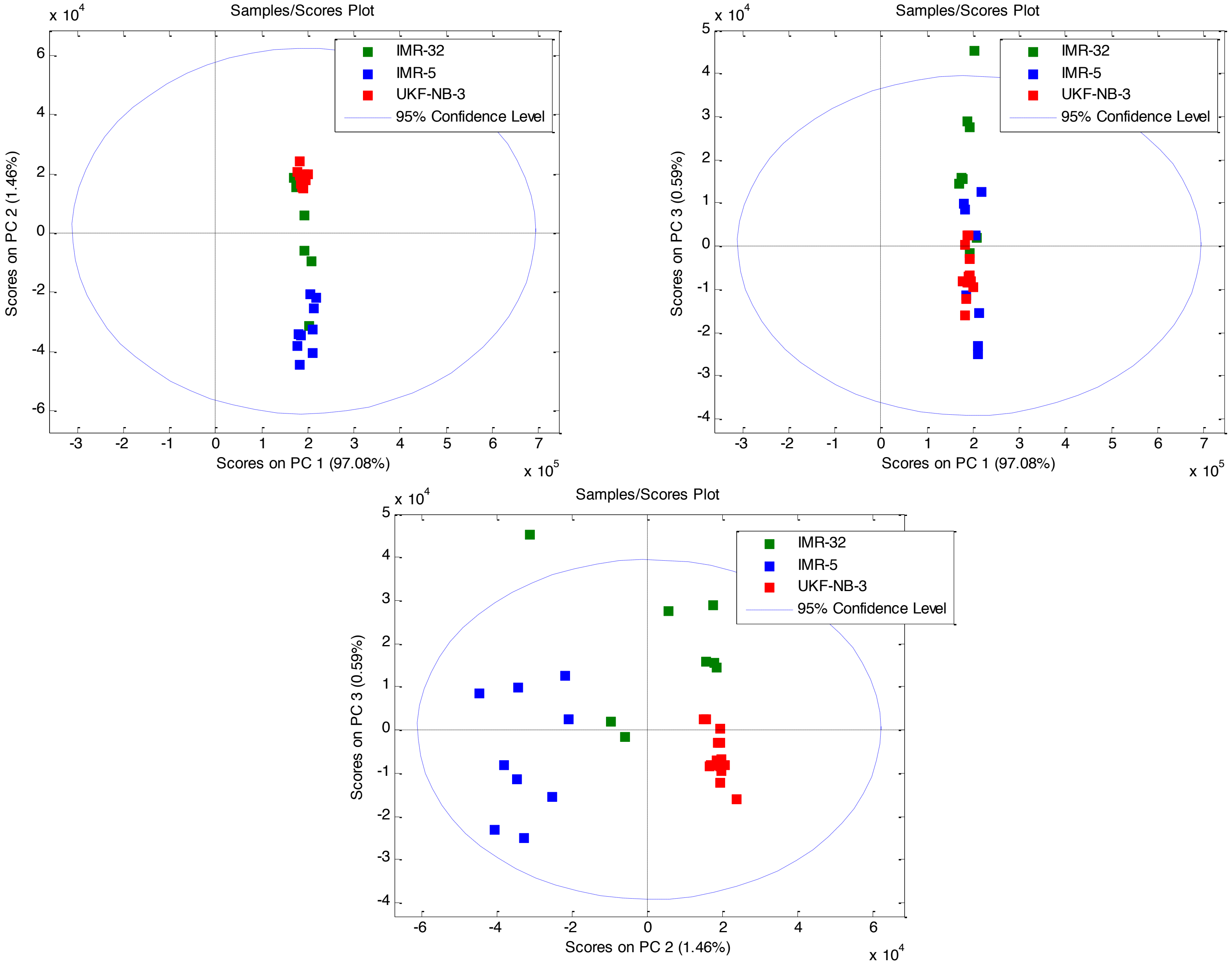

Samples/Scores Plot
x 10^4
Scores on PC 2 (1.46%)
Scores on PC 1 (97.08%)
x 10^5
IMR-32
IMR-5
UKF-NB-3
95% Confidence Level
Samples/Scores Plot
x 10^4
Scores on PC 3 (0.59%)
Scores on PC 1 (97.08%)
x 10^5
IMR-32
IMR-5
UKF-NB-3
95% Confidence Level
Samples/Scores Plot
x 10^4
Scores on PC 3 (0.59%)
Scores on PC 2 (1.46%)
x 10^4
IMR-32
IMR-5
UKF-NB-3
95% Confidence Level

Figure S4. Comparison of intact cell MALDI-ToF mass spectrometry analysis data derived from the cell lines IMR-32, IMR-5, and UKF-NB-3 by principal component (PC) analysis. The comparisons PC1 vs. PC2, PC1 vs. PC3, and PC2 vs. PC3 are presented.

Figure S5

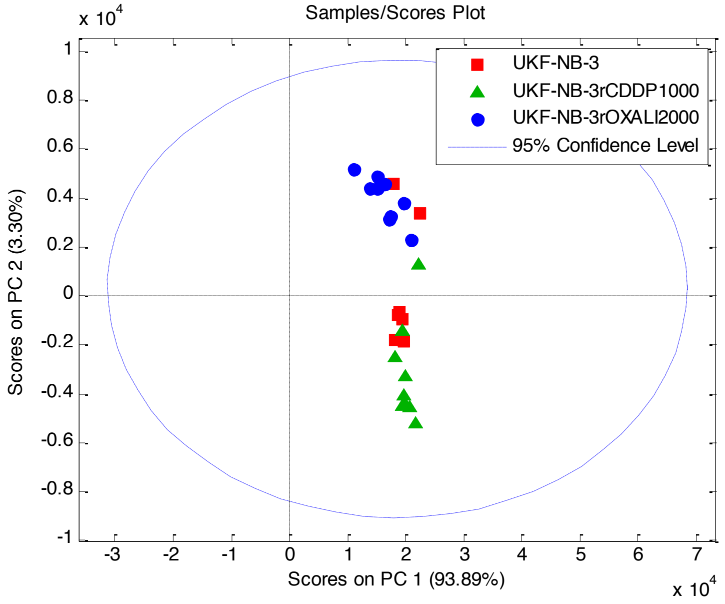
Samples/Scores Plot
x 10^4
Scores on PC 2 (3.30%)
Scores on PC 1 (93.89%)
x 10^4
UKF-NB-3
UKF-NB-3rCDDP1000
UKF-NB-3rOXALI2000
95% Confidence Level

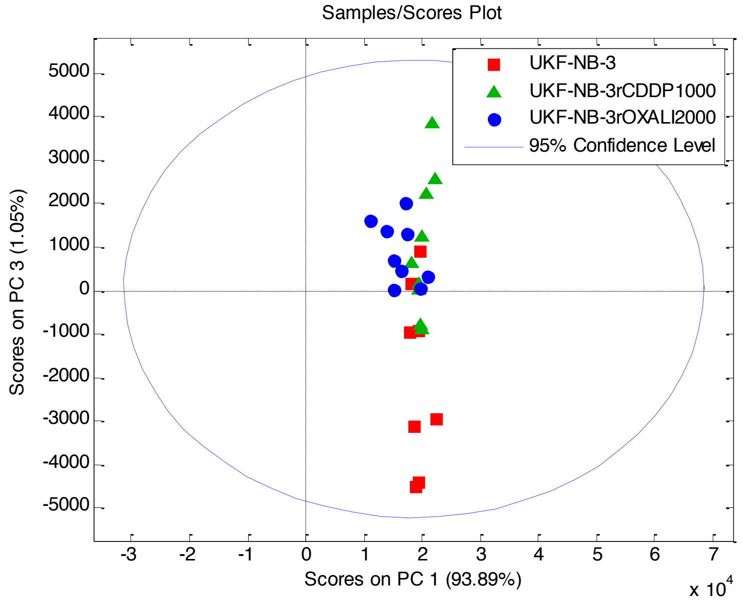
Samples/Scores Plot
Scores on PC 3 (1.05%)
Scores on PC 1 (93.89%)
x 10^4
UKF-NB-3
UKF-NB-3rCDDP1000
UKF-NB-3rOXALI2000
95% Confidence Level

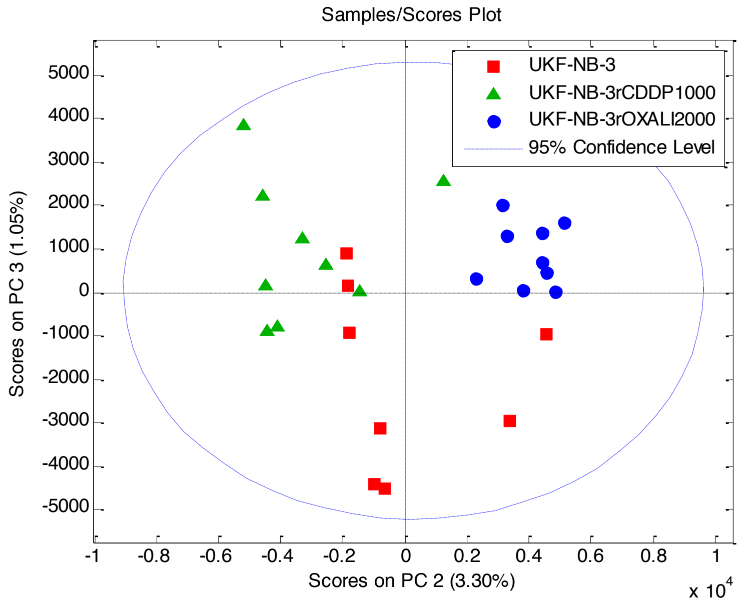
Samples/Scores Plot
Scores on PC 3 (1.05%)
Scores on PC 2 (3.30%)
x 10^4
UKF-NB-3
UKF-NB-3rCDDP1000
UKF-NB-3rOXALI2000
95% Confidence Level

Figure S5. Comparison of intact cell MALDI-ToF mass spectrometry analysis data derived from the cell line UKF-NB-3 and its drug-adapted sublines by principal component (PC) analysis. The comparisons PC1 vs. PC2, PC1 vs. PC3, and PC2 vs. PC3 are presented.

Figure S6

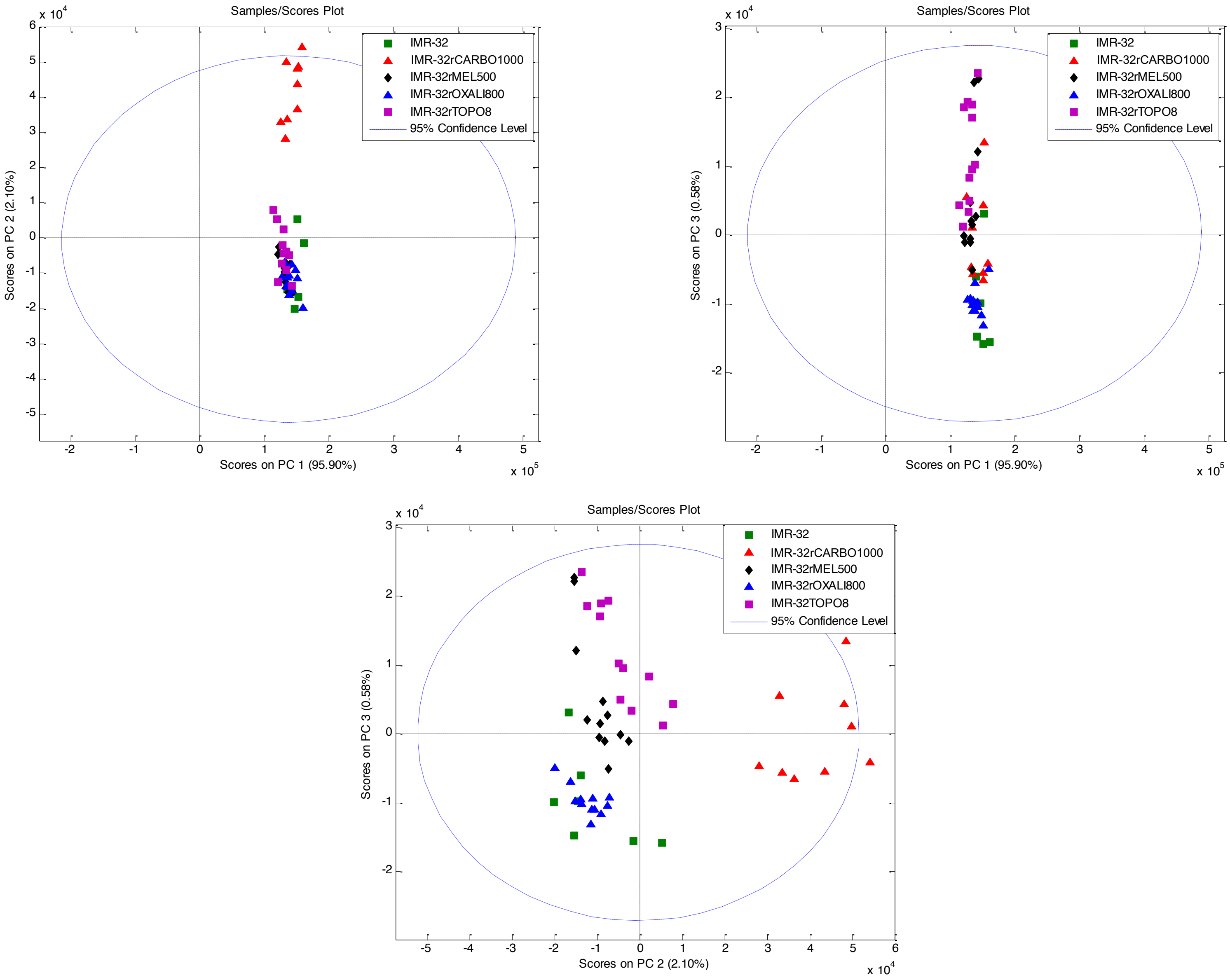

Samples/Scores Plot
Scores on PC 1 (95.90%)
Scores on PC 2 (2.10%)
x 10^5
x 10^4
IMR-32
IMR-32rCARBO1000
IMR-32rMEL500
IMR-32rOXALI800
IMR-32rTOPO8
95% Confidence Level
Samples/Scores Plot
Scores on PC 1 (95.90%)
Scores on PC 3 (0.58%)
x 10^5
x 10^4
IMR-32
IMR-32rCARBO1000
IMR-32rMEL500
IMR-32rOXALI800
IMR-32rTOPO8
95% Confidence Level
Samples/Scores Plot
Scores on PC 2 (2.10%)
Scores on PC 3 (0.58%)
x 10^4
x 10^4
IMR-32
IMR-32rCARBO1000
IMR-32rMEL500
IMR-32rOXALI800
IMR-32TOPO8
95% Confidence Level

Figure S6. Comparison of intact cell MALDI-ToF mass spectrometry analysis data derived from the cell line IMR-32 and its drug-adapted sublines by principal component (PC) analysis. The comparisons PC1 vs. PC2, PC1 vs. PC3, and PC2 vs. PC3 are presented.

Figure S7

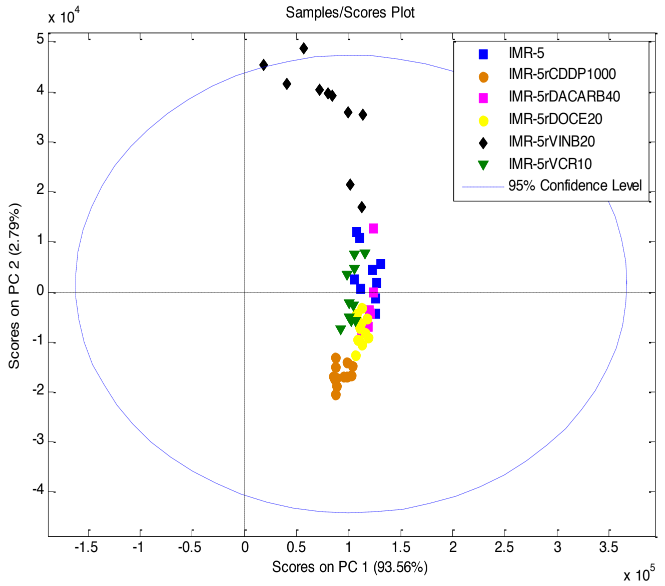
Samples/Scores Plot
x 10^4
Scores on PC 2 (2.79%)
Scores on PC 1 (93.56%)
x 10^5
IMR-5
IMR-5rCDDP1000
IMR-5rDACARB40
IMR-5rDOCE20
IMR-5rVINB20
IMR-5rVCR10
95% Confidence Level

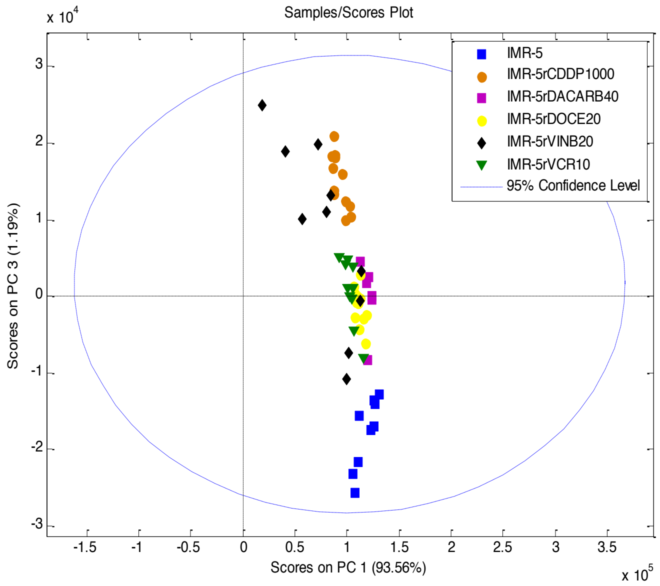
Samples/Scores Plot
x 10^4
Scores on PC 3 (1.19%)
Scores on PC 1 (93.56%)
x 10^5
IMR-5
IMR-5rCDDP1000
IMR-5rDACARB40
IMR-5rDOCE20
IMR-5rVINB20
IMR-5rVCR10
95% Confidence Level

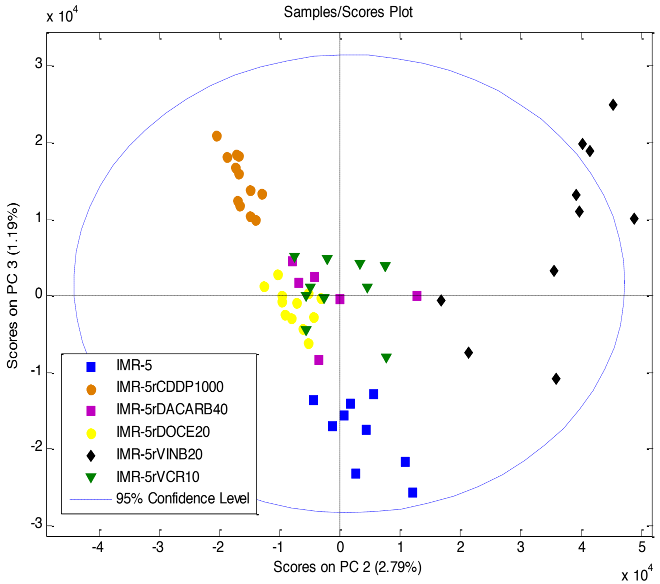
Samples/Scores Plot
x 10^4
Scores on PC 3 (1.19%)
Scores on PC 2 (2.79%)
x 10^4
IMR-5
IMR-5rCDDP1000
IMR-5rDACARB40
IMR-5rDOCE20
IMR-5rVINB20
IMR-5rVCR10
95% Confidence Level

Figure S7. Comparison of intact cell MALDI-ToF mass spectrometry analysis data derived from the cell line IMR-5 and its drug-adapted sublines by principal component (PC) analysis. The comparisons PC1 vs. PC2, PC1 vs. PC3, and PC2 vs. PC3 are presented.

Table S1. Short tandem repeat (STR) profiles of project cell lines**. Loci that differ from those of the parental cell line are highlighted in yellow.**

Cell line	D5	D5'	D13	D13'	D7	D7'	D16	D16'	vWA	vWA'	TH01	TH01'	TPOX	TPOX'	CSF1	CSF1'	Amel	Amel'
UKF-NB-3*	11	11	11	11	10	11	13	13	15	18	9	9.3	8	8	10	11	X	X
UKF-NB-3rCDDP1000	11	11	11	11	10	11	13	13	15	18	9	9.3	8	8	10	11	X	X
UKF-NB-3rOXALI2000	11	11	11	11	10	11	13	13	15	18	9	9.3	8	8	10	11	X	X
	D5	D5'	D13	D13'	D7	D7'	D16	D16'	vWA	vWA'	TH01	TH01'	TPOX	TPOX'	CSF1	CSF1'	Amel	Amel'
IMR-32 reference*	11	12	9	9	9	10	8	8	15	15	7	9.3	11	11	11	12	X	Y
IMR-32	11	12	9	9	9	10	8	8	15	15	7	9.3	11	11	11	12	X	Y
IMR-32rCARBO1000	11	12	9	9	9	10	8	8	15	15	7	9.3	11	11	11	12	X	X
IMR-32rMEL500	11	12	9	9	9	10	8	8	15	15	7	9.3	11	11	11	12	X	Y
IMR-32rOXALI800	11	12	9	9	9	10	8	8	15	15	7	9.3	11	11	11	13	X	Y
IMR-32rTOPO8	11	12	9	9	9	10	8	8	15	15	7	9.3	11	11	11	12	X	Y
	D5	D5'	D13	D13'	D7	D7'	D16	D16'	vWA	vWA'	TH01	TH01'	TPOX	TPOX'	CSF1	CSF1'	Amel	Amel'
IMR-5 reference*	11	12	9	9	9	10	8	8	15	15	7	9.3	11	11	11	12	X	Y
IMR-5	11	12	9	9	9	10	8	8	15	15	7	9.3	11	11	11	12	X	Y
IMR-5rCDDP1000	11	12	9	9	9	10	8	8	15	15	7	9.3	11	11	11	12	X	Y
IMR-5rDACARB40	11	12	9	9	9	10	8	8	15	15	7	9.3	11	11	11	12	X	X
IMR-5rDOCE20	11	12	9	9	9	10	8	8	15	15	7	9.3	11	11	11	12	X	Y
IMR-5rVINB20	11	12	9	9	9	9	8	8	15	15	7	9.3	11	11	11	12	X	X
IMR-5rVCR10	11	12	9	9	9	10	8	8	15	15	7	9.3	11	11	11	12	X	Y

* references: UKF-NB-3, no reference available in databases; IMR-32, DSMZ (ACC 165); IMR-5, D5-Amel (Sanger Institute)/ D3-D2 (indicated in grey) DSMZ (ACC 165)

Table S1. Short tandem repeat (STR) profiles of project cell lines**. Loci that differ from those of the parental cell line are highlighted in yellow.**

Cell line	D3	D3`	D21	D21`	D18	D18`	PentaE	PentaE	PentaD	PentaD	D8	D8`	FGA	FGA`	D19	D19`	D2	D2`
UKF-NB-3*	15	18	29	33.2	16	18	12	12	8	15	9	10	19	25	14	14	16	21
UKF-NB-3rCDDP1000	15	18	29	33.2	16	18	12	12	8	15	9	10	19	25	14	14	16	21
UKF-NB-3rOXALI2000	15	18	29	33.2	16	18	12	12	8	15	9	10	19	25	14	14	16	21
	D3	D3`	D21	D21`	D18	D18`	PentaE	PentaE	PentaD	PentaD	D8	D8`	FGA	FGA`	D19	D19`	D2	D2`
IMR-32 reference*	16	16	30	31	12	15	7	15	11	12	13	13	21	24	14	15	23	24
IMR-32	16	16	30	31	12	15	7	15	11	12	13	13	21	24	14	15	23	24
IMR-32rCARBO1000	16	16	30	31	12	15	7	15	11	12	13	13	21	24	14	15	23	24
IMR-32rMEL500	16	16	30	31	12	15	7	15	11	12	13	13	21	24	14	15	23	24
IMR-32rOXALI800	16	17	30	31	12	15	7	15	11	12	13	13	21	24	14	15	23	24
IMR-32rTOPO8	16	16	30	31	12	15	15	15	11	12	13	13	21	24	14	15	23	24
	D3	D3`	D21	D21`	D18	D18`	PentaE	PentaE	PentaD	PentaD	D8	D8`	FGA	FGA`	D19	D19`	D2	D2`
IMR-5 reference*	16	16	30	31	12	15	7	15	11	12	13	13	21	24	14	15	23	24
IMR-5	16	16	30	31	12	15	15	15	11	12	13	13	21	24	14	15	23	24
IMR-5rCDDP1000	16	16	30	31	12	12	15	15	11	12	13	13	21	24	14	15	23	24
IMR-5rDACARB40	16	16	30	31	12	12	15	15	11	12	13	13	21	24	14	15	23	24
IMR-5rDOCE20	16	16	30	32	12	15	15	15	11	12	13	13	21	24	14	15	23	24
IMR-5rVINB20	16	16	30	31	15	15	15	15	11	12	13	13	24	24	14	15	23	24
IMR-5rVCR10	16	16	30	31	12	15	15	15	11	12	13	13	21	24	14	15	23	24

* references: UKF-NB-3, no reference available in databases; IMR-32, DSMZ (ACC 165); IMR-5, D5-Amel (Sanger Institute)/ D3-D2 (indicated in grey) DSMZ (ACC 165)
